# Development of an All Solid State Battery Incorporating Graphene Oxide as Proton Conductor

**DOI:** 10.1002/gch2.201700054

**Published:** 2017-08-01

**Authors:** Yuta Shudo, Md. Saidul Islam, Mohammad Razaul Karim, Nurun Nahar Rabin, Kosuke Wakata, Ryo Ohtani, Masaaki Nakamura, Leonard F. Lindoy, Shinya Hayami

**Affiliations:** ^1^ Department of Chemistry Graduate School of Science and Technology Kumamoto University 2‐39‐1 Kurokami Chuo‐ku Kumamoto 860‐8555 Japan; ^2^ Department of Chemistry School of Physical Sciences Shahjalal University of Science and Technology Sylhet 3114 Bangladesh; ^3^ School of Chemistry The University of Sydney NSW 2006 Australia; ^4^ Institute of Pulsed Power Science (IPPS) Kumamoto University 2‐39‐1 Kurokami Chuo‐ku Kumamoto 860‐8555 Japan

**Keywords:** graphene oxide, solid electrolyte, solid state battery

## Abstract

Graphene oxide (GO) shows high proton conductivity (≈10^−4^ Scm^−1^), excellent mechanical stability, and electrical insulation property, which makes it an ideal candidate for use as a proton conducting solid state electrolyte. The prospects of using GO as single phase solid electrolyte in an all solid battery is presented herein. A battery with the cell configuration: Zn + ZnSO_4_•7H_2_O + graphite (anode) || GO (electrolyte) || MnO_2_ + graphite (cathode) is fabricated. Cyclic voltammetry confirms its rechargeable nature. The respective discharge capacity and power density of the cell are 360 μAh and 19.5 mW kg^−1^ at a constant current drain of 3 μA under the experimental conditions employed. GO based proton conductors are cleaner and cheaper than other solid electrolytes. The current study strongly suggests that GO can be used as a practical and beneficial component in solid state battery applications with low energy feedback.

## 1 Introduction

Solid state batteries (SSBs) with their special electrochemical and structural properties such as low leakage current, no reaction between electrolyte and electrode, faster charging, high energy density (in terms of volume), increased cycle life (up to 10 years), and nonflammability are becoming increasingly important in green energy technology and are expected to supersede conventional Li‐ion batteries (LIBs) in the near future.^1^, ^2^ However, the major drawback of this technology is the slower diffusion of ions through the solid electrolyte (SE) compared to the liquid electrolytes employed in LIBs. SEs have been viewed as the key component for improving the electrochemical performance of SSBs. As a result, the development of suitable SEs is being actively researched in an attempt to enhance the electrochemical energy output of SSBs.^3^ In general, two types of SE based on solid sulfides and solid oxides have been employed in SSBs. The sulfides have higher ionic conductivity than the oxides. On the other hand, the chemical stabilities of the oxides are much higher than those which occur for the sulfides.^4^ Up to the present, many attempts have been undertaken to develop all solid‐state batteries with Li^+^ conduction through SEs. The quest for such batteries became popular due to their ease of preparation, smooth operation over a wide temperature range, high stabilities, and safety.^1^, ^5^, ^6^, ^7^ Compared with Li^+^, H^+^ ions with their small ionic radius show better intercalation into the layered structure of cathodes. In addition, batteries having H^+^ ions as the current carrier lead to safer operation and lower cost. As a consequence, proton conducting electrolyte materials are theoretically quite important for the development of more efficient SSBs.^8^ The most basic properties of a material to function as H^+^ conducting SE are: (i) it should act as an electrical insulator that inhibits conduction of electrons between anode and cathode; that is, act as a separator, (ii) it should act as a fast proton conductor, and (iii) it should have a high degree of chemical stability. Graphene oxide (GO) possesses all of these essential properties and have been anticipated to be a promising candidate for the above battery application. High proton conductivities of GO and some of its hybrids as well as some of its derivatives have been reported in recent years.^9^, ^10^, ^11^, ^12^ GO contains various hydrophilic functional groups, which are responsible for proton conduction. Over the past decade, GO and its hybrids have been studied for many applications including as capacitor electrodes, in sensors, as well as in semiconductor and biomedical applications because of their outstanding adaptability.^13^, ^14^, ^15^ In this study, we have focused on the use of GO as a solid electrolyte in SSBs.

**Scheme 1:**
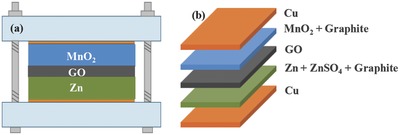
a) Design of solid state primary battery using GO electrolyte. b) Respective arrangement of cathode, anode, solid electrolyte, and current collector.

All reagents used in the study were of analytical grade and were used without further purification. GO was prepared by a modified hammers' method (for the detailed procedure see the Experimental Section).^16^ GO solution was prepared by dispersing graphite oxide in distilled water employing ultrasonication for 2 h followed by removal of insoluble matter by centrifugation. The GO solution was filtered using a nylon membrane under reduced pressure to produce GO paper. The structure and morphology of the latter were characterized by X‐ray photoelectron spectroscopy (XPS), atomic force microscopy (AFM), scanning electron microscopy (SEM), and Raman spectroscopy. The ionic conductivities of the solid electrolytes (namely, the bulk GO and GO paper samples) were measured by a four probe AC method using an impedance/gain phase analyzer (Solartron 1260) over the frequency range of 1–10^6^ Hz.^26^ Measurements were performed under controlled temperature and humidity using an incubator (SH‐221, ESPEC). The detail configurations of bulk GO and GO paper cells for proton conductivity measurement were shown in Experimental Section and Figure S1 (Supporting Information). The battery cell was prepared by sandwiching GO paper (incubated at 90% relative humidity at room temperature for 5 h) between the cathode and anode (**Scheme** **1**). Anode and cathode plates were prepared employing zinc:zinc sulfate:graphite in the ratio of 1:1:2 and manganese oxide:graphite in 1:1 ratio, respectively. For the cathode, 0.1 g Zn, 0.1 g ZnSO_4_ and 0.2 g graphite were mixed thoroughly using a porcelain mortar. The mixture was then pressed (40 MPa) into a pellet using a mini press (Riken Seiki Co. Ltd. P‐016B‐027B). The cathode pellet was made by using 0.2 g MnO_2_ and 0.2 g graphite using a similar process. The thickness of anode and cathode were 1.45 and 1.36 mm, respectively. Cu foil was used as the current collector. Electrochemical characteristics of the battery were measured with a potentiostat and a function generator (IVIUM TECHNOLOGES, Compact Stat) at room temperature. The battery cell was discharged and charged under different load conditions ranging from 3 to 20 μA and the cell potentials were monitored as a function of time. Cyclic voltammetry was conducted in the potential range of 0.01−2.75 V at a scan rate of 0.1 mV s^−1^.

**Figure 1:**
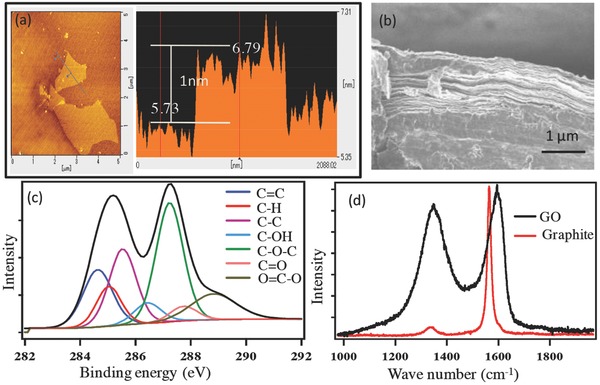
Characterization of GO. a) AFM image, b) SEM image of GO paper (cross section), c) C 1s XPS spectra of GO, and d) Raman spectra of graphite and GO.

Aspects of the structural morphology of GO are presented in **Figure** **1**. AFM analysis (Figure 1a) confirms the formation of pristine graphene oxide nanosheets with 1 nm thickness; this is consistent with the typical morphology reported in previous studies.^17^ The SEM image (Figure 1b) displays the layered structure. Figure 1c presents the XPS C 1s spectra of GO and complies with a previous report.^18^ Characteristic peaks associated with particular oxygenous functional groups were observed. For example, the existence of peaks for epoxide groups (—O—) at 287.2 eV, hydroxyl groups (—OH) at 286.5 eV, carbonyl groups (—C=O) at 287.8 eV, and carboxyl (—COOH) groups at 289.9 eV confirm the successful oxidation of graphite to GO. Successful conversion of graphite to GO with sp^2^ → sp^3^ C conversion was further confirmed from the Raman spectra (Figure 1d).

The existence of D and G bands near 1350 and 1580 cm^−1^ is characteristic of carbon based materials. These D and G bands are responsible for the breathing mode of sp^3^ C atoms (A1g) and the in‐plane bond stretching motion of pairs of sp^2^ C atoms (E_2_g), respectively.

**Figure 2:**
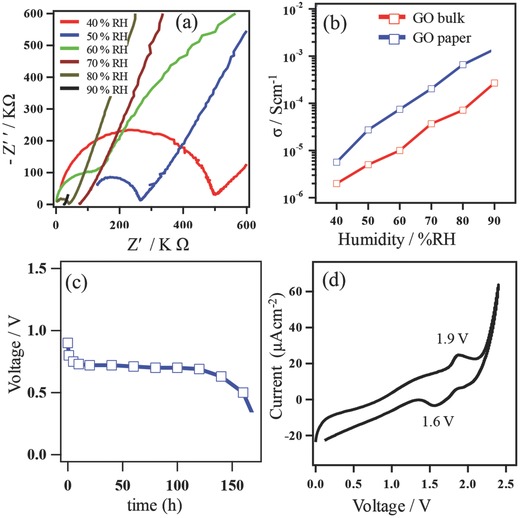
Electrochemical performance. a) Nyquist plots of bulk GO at room temperature and 40–90% RH. b) Proton conductivities calculated from the Nyquist plots. c) Discharge properties (voltage vs time) for the battery obtained at 3 μA current drain. d) Cyclic voltammogram obtained at a scan rate 0.1 mV s^−1^.

Furthermore, the *I*_D_/*I*_G_ peak ratio signifies the ratio between sp^3^ and sp^2^ hybridized carbon sites in graphitic materials. The *I*_D_/*I*_G_ values of 0.134 and 0.973 for graphite and GO, respectively, thus confirm the presence of more sp^3^ character for GO than for graphite. GO has randomly allocated nonconductive sp^3^ carbon sites that are responsible for abolishing the electrical conductivity.^19^ Therefore, in contrast to graphite, GO is an electronic insulator. **Figure** **2**a shows the Nyquist plots of bulk GO pellet with respect to relative humidity (RH) at room temperature. The track formed by real (Z′) and imaginary (‐Z″) parts of the impedance was found to “fit” to distorted semicircular curves where the diameters of the semicircles represent resistance. Conductivity was calculated using: σ = *L*/(*R* × *A*), where *L* is thickness of the pellet, *R* is the resistance, and *A* is the surface area of the interface between the pellet and the electrodes. The appearance of a second semicircle indicates that the conductivity is driven by protons. Proton oriented conductivity was further confirmed by the fact that the conductivity of H_2_O‐humidified GO was ≈1.3 times higher than that of a D_2_O‐humidified sample.^20^ The respective conductivity values given in Figure 2b show that proton conductivity of bulk GO increases gradually with respect to RH at room temperature. At 40 and 90% RH, the proton conductivity values are 1.4 × 10^−6^ and 3.7 × 10^−4^ Scm^−1^, respectively.

As shown in Figure 2b, GO paper exhibited about one order higher proton conductivity than that of bulk GO. For GO paper, the σ value represents the in‐plane conductivity whereas, in bulk GO, the particles are randomly ordered and σ should be a combination of both the in‐plane and through‐plane conductivities. Since in‐plane conductivity is much higher than that of through‐plane conductivities, GO paper shows higher conductivity than bulk GO. Moreover, numerous reports suggest that proton conduction along nanoscale films is usually higher than that in bulk compounds.^21^ Figure 2c shows the discharge profiles for the battery cell with a test current drain of 3 μA. The voltage versus time curve exhibits a rapid fall initially and then remains stable for ≈120 h and then the curve again decreases rapidly; the open circuit voltage value of the cell is ≈0.7 V. Corresponding discharge capacity and power density have been calculated to be about 360 μAh and 19.5 mW kg^−1^, respectively (for details of the calculations see the Supporting Information). Figure 2d shows the cyclic voltammogram (CV) profile of the battery cell over 0–2.5 V at a scan rate of 0.1 mV s^−1^ which is a typical rate for a reversible cell.^22^ The anodic CV sweep shows a peak at 1.9 V while the cathodic sweep displays a peak at 1.6 V. These peaks arise from the respective oxidation and reduction process of Zn and MnO_2_.^23^ Figure S2 (Supporting Information) displays the recharge ability of the cell. The cell was charged and discharged using a constant current of 20 μA. It can be seen that the cell regained its initial potential value after recharging. The cycling ability has been studied up to eleven cycles with no significant loss in potential being observed. The overall redox reaction for this system is as follows^24^

At anode

(1) $$\mathrm{Zn}\;\;\to \;\;{\mathrm{Zn}}^{2+}+2\;e^{-}$$

At cathode

(2) $$2\;e^{-}\;\;+\;\;{\mathrm{MnO}}_{2}+4\;H^{+}\;\;\to \;\;{\mathrm{Mn}}^{2+}+2\;H_{2}O$$

Overall reaction

(3) $$\mathrm{Zn}\;\;+\;\;{\mathrm{MnO}}_{2}+4\;H^{+}\;\;\to \;\;{\mathrm{Zn}}^{2+}+{\mathrm{Mn}}^{2+}+2\;H_{2}O$$

Clearly, SSBs with SEs have a variety of advantages over conventional batteries made with liquid electrolytes. In order to develop a practical high‐performance SSB, a very high proton conductive solid electrolyte is required. The introduction of oxygen functional groups onto GO usually generates an interlayer distance of ≈8 Å, which was confirmed in the present study from the corresponding powder X‐Ray diffraction (PXRD) pattern (Figure S3, Supporting Information).^25^ Water molecules can form H‐bonds with such oxygenated functional groups on both the faces of GO and act to support fast proton conduction.^26^ In the present study, based on such high proton conductivity, our aim has been to develop SSBs using GO SEs. Moreover, as thin film GO is able to exhibit proton conductivity without any electronic conductivity, it also appeared feasible to develop a thin film (nm size) battery. The energy output (360 μAh) that we obtained from our cell is comparable with other reported solid state batteries.^27^, ^28^, ^29^ For example, Agrawal et al. fabricated an all solid state, proton‐conducting polymeric battery with the cell configuration: Zn + ZnSO_4_•7H_2_O (anode) || polyethylene oxide (PEO)+ NH_4_HSO_4_ + SiO_2_ || MnO_2_ + C (cathode), where the observed discharge capacity was 110 μAh.^28^ In a similar study, Lakshmi and Chandra reported a proton conducting battery where the solid electrolyte was obtained by dispersing heteropolyacid hydrates (a mixture of phosphotungstic acid and phosphomolybdic acid (PMA) into insulating Al_2_O_3_, Al_2_(SO_4_)_3_.16H_2_O, and (NH_4_)_10_W_12_O_4_•12H_2_O. This battery with cell configuration: Zn + ZnSO_4_•7H_2_O + MH*_x_* (anode) || PMA + APT || PbO_2_ + V_2_O_5_ (cathode) exhibited a discharge capacity of 0.53 mAh.^29^ The energy output can be further increased by reducing the interfacial resistance between the electrode and the electrolyte. Surface modification techniques such as ball‐milling, surface coating of active material particles with SE thin films, and softening glass electrolytes are the reported effective means for obtaining enhanced contact between the electrode and the electrolyte. By such means minimization of interfacial resistance, with improved battery performance, can be achieved.^30^ In view of this, we expect that in near future it will be possible to optimize the interfacial resistance of the cell to obtain high‐performance rechargeable solid state batteries based on GO.

## 2 Conclusion

In conclusion, the fabrication of an all solid state battery has been carried out employing GO, Zn, and MnO_2_ as the solid electrolyte, anode and cathode, respectively. The “as‐prepared” solid state system was evaluated under constant current drain conditions at room temperature. The measured discharge capacity was 360 μAh. Although in its present state, the current density of this solid state battery is somewhat lower than that of a Li‐ion battery, the ability to achieve a very thin shape, together with the use of cheap and nontoxic solid electrolytic components clearly points the way to possible practical application in near future.

## 3 Experimental Section

*Preparation of GO*: Graphite (1.0 g) and NaNO_3_ (1.0 g) were dissolved in conc. H_2_SO_4_ (48 mL) and the mixture was stirred for 30 min in a round bottom flask while the temperature was maintained at 0 °C using an ice bath. KMnO_4_ (3.0 g) was then added to this solution with stirring and maintained at 35 °C. After 40 min, deionized water (400 mL) and 30% H_2_O_2_ (12 mL) were added to the solution. Then the solid was separated by centrifugation and washed with 5% HCl solution, followed by washing with deionized water until the pH of the solution became neutral. The graphite oxide solid was dried overnight at 60 °C. GO solution was prepared by dispersing graphite oxide in distilled water employing ultrasonication for 2 h followed by removal of insoluble matter by centrifugation.

*Preparation of Cells for Proton Conductivity Measurement*: In order to measure through plane conductivity, dried GO were compressed into pellet with a diameter of 1.3 cm and thicknesses of 1 mm. Both sides of pellet were attached to gold wire (50 μm diameter, Tanaka Kikinzoku Kogyo) with gold paste (Figure S1a, Supporting Information). In case of measuring in plane proton conductivity, GO paper was used. Two gold electrodes were attached in the same side of GO paper while maintained a distance of 1 cm (Figure S1b, Supporting Information).

## Conflict of Interest

The authors declare no conflict of interest.

## Supporting information

Supplementary
