# Supplementary material for: Development of an All Solid State Battery Incorporating Graphene Oxide as Proton Conductor
Source: Glob Chall. 2017 Aug 1;1(6):1700054. doi: 10.1002/gch2.201700054 (PMC6607141; doi:10.1002/gch2.201700054)
Supplement: Supplementary file 1 — Supplementary [file GCH2-1-1700054-s001.pdf]

Copyright WILEY-VCH Verlag GmbH & Co. KGaA, 69469 Weinheim, Germany, 2017.

Supporting Information

for *Global Challenges,* DOI: 10.1002/gch2.201700054

Development of an All Solid State Battery Incorporating Graphene Oxide as Proton Conductor

*Yuta Shudo, Md. Saidul Islam, Mohammad Razaul Karim, Nurun Nahar Rabin, Kosuke Wakata, Ryo Ohtani, Masaaki Nakamura, Leonard F. Lindoy, and Shinya Hayami**

Supporting Information

Title Development of an all solid state battery incorporating graphene oxide as proton conductor

*Yuta Shudo, Md. Saidul Islam, Mohammad Razaul Karim, Nurun Nahar Rabin, Kosuke Wakata, Ryo Ohtani, Masaaki Nakamura, Leonard F. Lindoy and Shinya Hayami**

Calculation of discharge capacity and power density:

Discharge Capacity (Ah) = (applied current × time in platitude region)
= 3µA× 120h
= 360 µA h

Power density (W/kg) = (applied current × voltage achieved)/ active materials in Kg
= (3µ A × 0.65V)/ .0001 Kg
= 1.95 µW/ 0.0001Kg
=19500 µW/Kg
= 19.5 mW/Kg

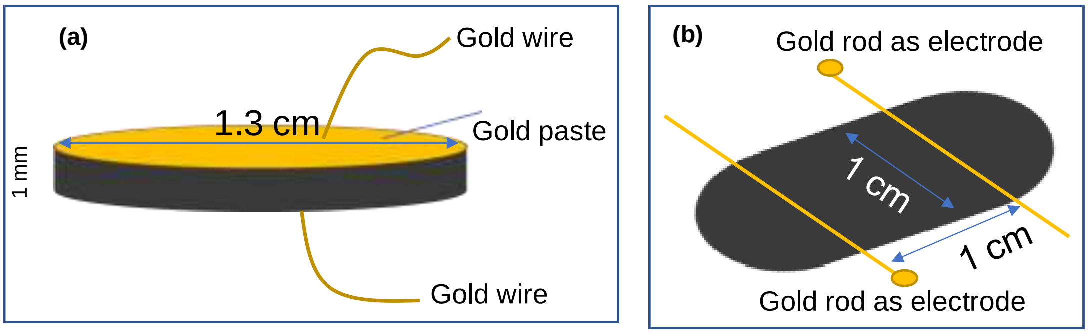

Figure S1: a) Cell configuration for measurement of GO bulk proton conductivity (through plane direction), b) Cell configuration for measurement of GO paper proton conductivity (in plane direction).

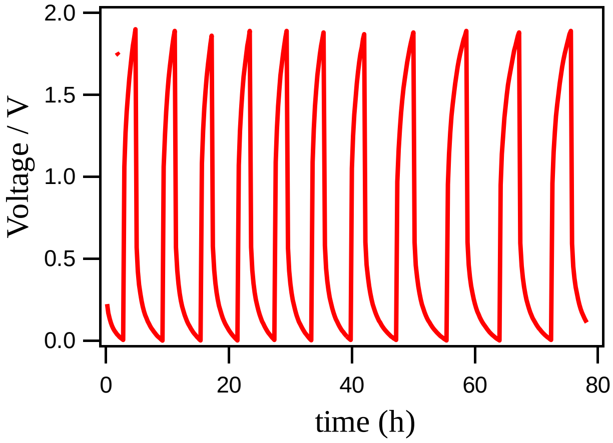

Figure S2: Charge-discharge properties of the battery obtained at a current drain of 20 μA.

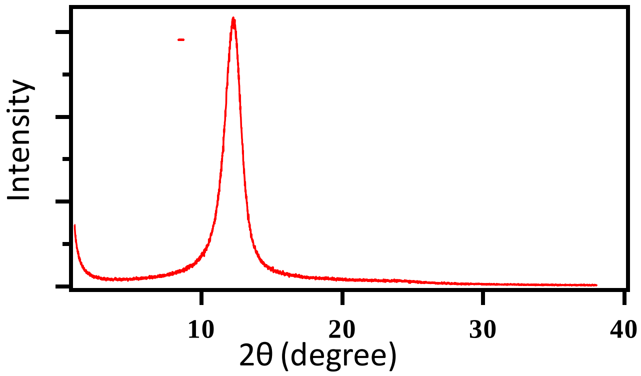

Figure S3: XRD pattern of GO paper
